# Effects of Proton Therapy on Cardiac Fibrosis, Calcium Homeostasis, and AQP4 Expression in Hypergravity-Exposed Rats

**DOI:** 10.3390/ijms26136326

**Published:** 2025-06-30

**Authors:** Hyewon Park, Bokyeong Park, Kyu-Sung Kim, Hyelim Park, Junbeom Park

**Affiliations:** 1 Department of Cardiology, College of Medicine, Ewha Womans University School of Medicine, Seoul 07804, Republic of Korea; kiko0615@ewha.ac.kr (H.P.); whitecat0804@naver.com (B.P.); 2 Department of Otorhinolaryngology-Head and Neck Surgery, Inha University School of Medicine, Incheon 22332, Republic of Korea; stedman@inha.ac.kr

**Keywords:** space environment stimulation, arrhythmia, proton therapy, fibrosis

## Abstract

Proton therapy is increasingly used to treat pediatric and adult brain tumors, but there is still uncertainty surrounding the biological effects of protons on the heart. Also, the molecular and functional responses to proton irradiation are still unknown. This study investigates the effect of protons on cardiac disease by comparing their effects on the hearts of rats exposed to hypergravity. A total of 20 Sprague Dawley rats were tested, including a group that was irradiated with 0.1 Gy of protons to the heart, a group exposed to hypergravity, a group exposed to both protons and hypergravity, and a control group. Changes in AQP4, calcium homeostasis, and fibrosis-related markers were investigated using Western blotting, immunohistochemistry, etc. The proton-irradiated group showed no changes compared to the control group. In rats exposed to hypergravity, the cardiac fibrosis markers TGF-ꞵ1, MMP9, and MMP2 were increased. On the other hand, the group exposed to hypergravity followed by proton irradiation tended to display a significant decrease in these markers. Along with reduced fibrosis-related markers, the consistent tendency was also confirmed in the cardiac calcium homeostasis-related proteins and AQP4 through Western blotting. In summary, our findings indicate that rats subjected to hypergravity experienced both cardiac hypertrophy and fibrosis, while proton therapy appeared to mitigate the effects of cardiac disease. These results suggest that proton therapy prevents heart disease triggered by hypergravity, providing insights for protecting astronauts’ cardiovascular health.

## 1. Introduction

Currently, radiation therapy is mainly used to treat cancer, and it is not common to treat heart disease itself with radiation. Also, radiation therapy is used to destroy cancer cells or inhibit their growth, and it is mainly aimed at curing cancer or alleviating symptoms. However, some studies show that such radiation therapy for managing cancer can affect the heart. For example, a study reported that radiation therapy can increase the risk of heart disease when patients with left-sided breast cancer are treated [1,2]. Despite such potential risks, recent studies suggest that adjusted radiation therapy may have potential benefits in improving symptoms of certain heart conditions. For example, a study from the University of Washington found that 5 Gy of low-dose radiation therapy may improve heart function in patients with heart failure [3]. The study suggests that radiation may improve heart function by reducing the number of inflammatory immune cells in the heart muscle. There is also a study that found that high-dose radio ablation can significantly reduce the frequency of arrhythmia episodes in patients with ventricular tachycardia, a serious arrhythmia. This method is used as a last resort for patients who have exhausted all existing treatment options and has been shown to improve survival and quality of life in the short term [4]. However, radiation therapy may have long-term negative effects on the heart, especially at high doses, which may increase the risk of heart disease [5]. Also, the molecular mechanism regarding radiation therapy for heart disease is still unclear. Therefore, it is difficult to say that radiation therapy guarantees a complete cure for heart disease at present, and it is likely to be used mainly for symptom relief and as an adjuvant treatment in certain situations.

Proton therapy is gaining increasing attention for its role in treating and preventing heart diseases. This innovative approach offers effective treatment while minimizing radiation exposure to the heart and surrounding tissues. This non-invasive approach may offer several advantages over traditional catheter ablation procedures. Recent research has shown that proton beam therapy can affect the electrophysiological properties of the myocardium and potentially be used to treat life-threatening arrhythmias under controlled exposure [6]. For instance, studies have observed changes such as decreased connexin-43 expression, reduced conduction velocity, and decreased bipolar electrogram amplitude following high-dose proton irradiation [6]. On the other hand, there are studies that demonstrate the potential therapeutic effects of low-dose proton exposure on tachyarrhythmias and cardiac remodeling [7,8,9]. These results from previous studies conducted under various conditions provide valuable insights into the effects and underlying mechanisms of proton treatment. Although proton therapy has recently emerged as a promising approach for enhancing precision and safety in the treatment of cardiac diseases, further research is required to evaluate its long-term effects and to support the expansion of its clinical applications in cardiology.

This study has the potential to yield significant insights into strategies for preserving cardiovascular health in astronauts and protecting cardiac function in patients undergoing radiation therapy. Our study provides crucial contributions in two key areas of investigation. The first regards astronaut cardiovascular health: Understanding how proton therapy might mitigate the cardiac effects of prolonged exposure to altered gravity environments, including repeated High-G training, launching and reentry [10,11], could be essential for space missions. This could contribute to maintaining the cardiovascular health of astronauts. The second complements the limitation of radiation therapy in terms of cardiac protection: for breast cancer patients receiving radiation treatment near the heart [1,12,13], this research might reveal new strategies to minimize cardiac damage and maintain heart function. Therefore, this study aims to induce cardiac hypertrophy in rats through hypergravity exposure and subsequently assess the efficacy of proton therapy for the prevention and treatment of cardiac disease. This approach could uncover novel methods for cardiac care in both space medicine and oncology.

## 2. Results

### 2.1. Cardiac Hypertrophy and Histological Findings

**Figure 1:**
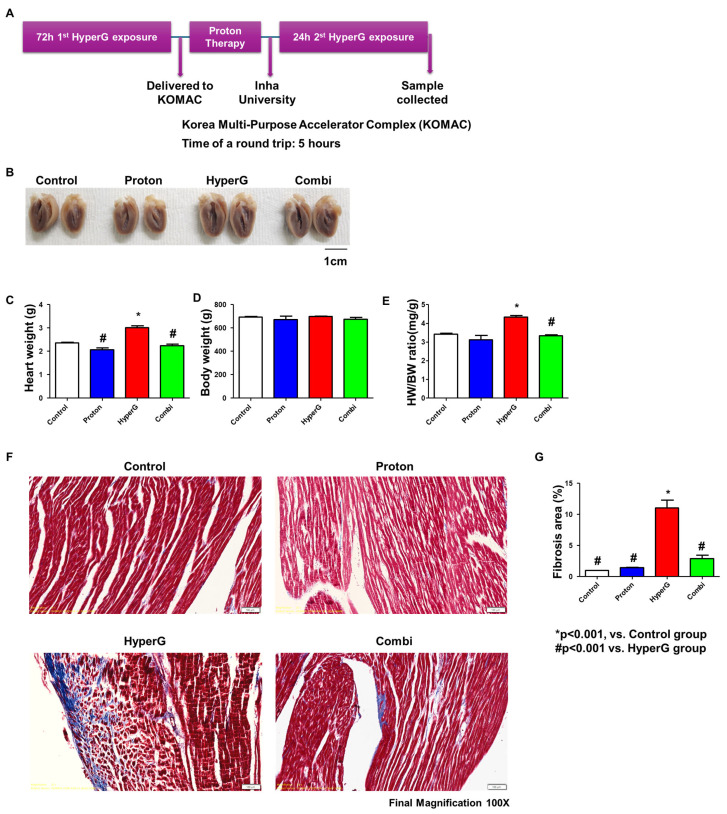
Gross and cardiac fibrosis histological analyses of the heart. (**A**) Animal experiment timeline. (**B**) Gross images of the hearts in each of the four groups. (**C**–**E**) Heart weight, body weight, and the ratio of the heart weight to the total body weight. (**F**) Images of cardiac fibrosis area indicated in blue for each of the four groups. (**G**) Quantification of the percentage of fibrotic area. * *p* < 0.001 vs. Control. Combi: HyperG group treated with Proton. # *p* < 0.001 vs. HyperG.

Figure 1A represents the timeline of animal experiment. In detail, the rats were exposed to HyperG (4G) and proton therapy, followed by 24 h HyperG exposure. The gross values of each of the four groups are presented in Figure 1B. The heart weights were 2.39 ± 0.1 g, 2.18 ± 0.1 g, 3.05 ± 0.1 g, and 2.34 ± 0.1 g in the Control, Proton, Hypergravity (HyperG), and Combination (HyperG + Proton; Combi) groups, respectively (Figure 1C). The body weights showed no significant differences between the four groups (Control 692.3 ± 9.7, Proton 670.8 ± 52.3, HyperG 696.5 ± 6.8, Combi 673.5 ± 25.2 g) (Figure 1D). The ratios of heart weight to body weight were 3.405 ± 0.091, 3.101 ± 0.405, 4.326 ± 0.135, and 3.326 ± 0.0.089, respectively (Figure 1E). The HyperG group had heavier heart weights than the Control, Proton, and Combi groups (*p* < 0.001). The ratio of heart weight to body weight was also larger in the HyperG group than in the Controls (*p* < 0.001). Figure 1F shows the Masson’s trichrome stain in rat hearts. HyperG rats showed increased levels of fibrosis compared with the Control group (1.3 ± 0.1 vs. 11.0 ± 2.4, *p* < 0.001), while the Combi group had less fibrosis than the HyperG group (11.0 ± 2.4 vs. 2.8 ± 1.1, *p* < 0.001) (Figure 1G). To summarize the results, cardiac hypertrophy and fibrosis were developed in rats exposed to HyperG, while the HW/BW ratio and fibrosis decreased in the Proton group.

### 2.2. Evaluation of Fibrosis Area in Rat Hearts Treated with Proton Therapy

**Figure 2:**
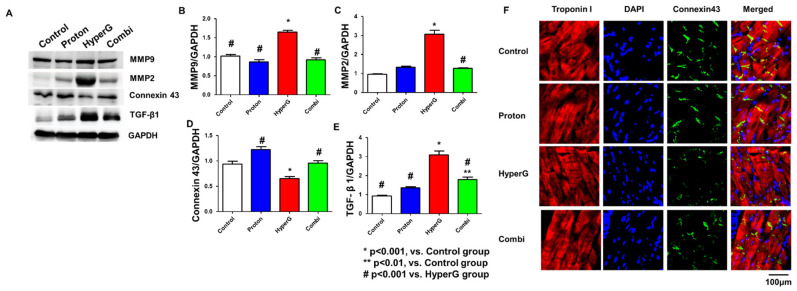
Increased cardiac fibrosis in high gravity. (**A**) Western blot analysis for MMP9, MMP2, Connexin43, and TGF-β1 in heart tissue from each group. (**B**–**E**) Levels of MMP9, MMP2, Connexin43, and TGF-β1 in heart tissue. (**F**) Immunofluorescence staining of Troponin I and Connexin43 in heart tissue of each group. Magnification 400×. Data are means ± SEMs. * *p* < 0.001 vs. Control. ** *p* < 0.01 vs. Control. # *p* < 0.001 vs. HyperG.

To assess fibrosis in rat hearts treated with proton therapy, we compared the levels of fibrosis-related proteins among the Control, Proton, HyperG, and Combi groups. The protein expression levels of MMP9, MMP2, TGF-β1, and Connexin43 were evaluated in atrial tissue homogenates (Figure 2A). Western blot results showed that MMP9 (1.6 ± 0.1-fold change), MMP2 (3.0 ± 0.2-fold change), and TGF-β1 (3.0 ± 0.1-fold change) were increased and Connexin 43 (0.6 ± 0.1-fold change) was decreased in the hearts of HyperG group rats compared with the Control group (*p* < 0.001). However, in the Combi group, MMP9 (0.9 ± 0.1-fold change), MMP2 (1.2 ± 0.1-fold change), and TGF-β1 (1.9 ± 0.1-fold change) were significantly reduced and Connexin 43 (0.9 ± 0.1-fold change) was recovered compared to the HyperG group (*p* < 0.001) (Figure 2B–E). In addition, immunofluorescence staining demonstrated that Connexin43 expression at the intercalated discs of cardiomyocytes was reduced in the HyperG group and restored in the Combi group, consistent with the Western blot results. This suggests that myocardial fibrosis was alleviated by proton treatment.

### 2.3. Increased p-CaMKII, RyR2, and p-PLB in HyperG Group

**Figure 3:**
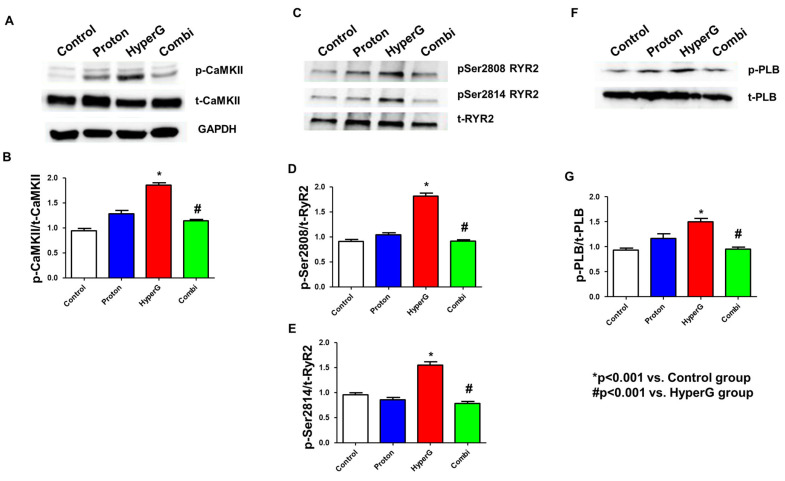
Changes in cardiac Ca^2+^-handling proteins in rat heart tissues. (**A**) Representative Western blots for Thr287-CaMKII phosphorylation, total CaMKII protein, and GAPDH expression in the heart tissues of the four groups. (**B**) Quantification of total CaMKII expression and relative Thr287 phosphorylation levels. (**C**) Representative Western blots of Ser2808 and Ser2814 phosphorylated RyR2 and total RyR2 expression in heart tissues. (**D**,**E**) Total RyR2 expression and Ser2808 and Ser2814 phosphorylation levels. (**F**) Representative Western blots for Thr17-PLB phosphorylation and PLB protein. (**G**) Total PLB expression and phosphorylation levels of Thr17. * *p* < 0.001 vs. Control. # *p* < 0.001 vs. HyperG. CaMKII: calmodulin-dependent protein kinase II, RyR2: ryanodine 2 receptor, PLB: phospholamban.

We assessed CaMKII, RyR2, and PLB expressions and relative phosphorylation levels by Western blotting. Figure 3A shows the Ca^2+^ handling protein assay in rat ventricular tissues. CaMKII and Thr287-CaMKII protein expressions were significantly increased in HyperG tissue lysates (1.0 ± 0.1 vs. 1.8 ± 0.1, *p* < 0.001). However, there was a significant decrease in the Combi group compared to the HyperG group (1.8 ± 0.1 vs. 1.1 ± 0.1, *p* < 0.001) (Figure 3B). With a slightly increased total RyR2, both Ser2808-RyR2 (0.9 ± 0.1 vs. 1.8 ± 0.1, *p* < 0.001) and Ser2814-RyR2 (1.0 ± 0.1 vs. 1.6 ± 0.1, *p* < 0.001) protein expressions were significantly increased in HyperG tissue lysates (Figure 3C–E). Moreover, the ratio of Thr17-PLB to total PLB, which increased in the HyperG group (Figure 3F–G), was restored by the Combi group (1.5 ± 0.1 vs. 0.9 ± 0.0, *p* < 0.001). To summarize the results, proton therapy recovered the calcium homeostasis in the hearts of rats exposed to HyperG through the alleviation of CaMKII, RyR2, and PLB phosphorylation.

### 2.4. Evaluation of AQP4 Gene and Protein Expression Levels

**Figure 4:**
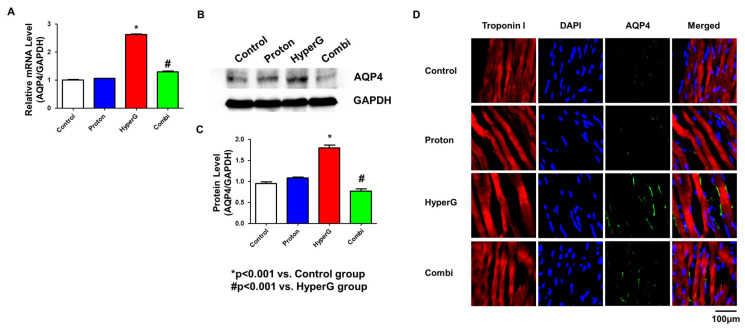
Changes in AQP4 mRNA and protein expression in heart tissue exposed to HyperG. (**A**) mRNA was extracted from heart tissues of the four groups and analyzed by qPCR. (**B**,**C**) Protein extracted from heart tissues of each group and analyzed by Western blot. (**D**) Expression analysis of Troponin I and AQP4 in heart tissues of the four groups by immunofluorescence staining. Magnification 400×. * *p* < 0.001 vs. Control. # *p* < 0.001 vs. HyperG.

In the qPCR test, the AQP4 mRNA expression level was found to be increased in the HyperG group compared with the Control group (1.0 ± 0.0 vs. 2.5 ± 0.1 compared with Control group, *p* < 0. 001) (Figure 4A). Meanwhile, the AQP4 expression level in the Combi group was significantly decreased compared with that in the HyperG group (2.5 ± 0.1 vs. 1.2 ± 0.1, *p* < 0.001). Similarly, in the Western blot analysis, it was significantly increased in the HyperG group compared to the Control group (1.0 ± 0.1 vs. 1.8 ± 0.1, *p* < 0.001) (Figure 4B,C). Additionally, in immunofluorescence staining, the expression level of AQP4 was relatively increased in the myocardial interstitium of the HyperG group. The expression level of AQP4 in the Combi group was significantly decreased compared to the HyperG group. To recapitulate the findings, the level of AQP4 mRNA and protein were upregulated in the hearts of rats exposed to HyperG and were reversed by proton therapy. This development demonstrates the potential importance of proton therapy in the adjustment of AQP4 levels.

## 3. Discussion

There are two critical contexts in this study: (1) the potential effects of proton treatment on cardiac health and (2) developing advanced cardiovascular protection strategies for astronauts. Therefore, the rationale behind this study is to identify changes in cardiac fibrosis, calcium-handling proteins, and AQP4 in rat models exposed to HyperG and proton therapy. SD rats were exposed to HyperG and then underwent proton therapy, followed by an additional HyperG exposure. As a result, rats exposed to HyperG showed increased heart weights, while those receiving proton therapy did not exhibit significant changes.

Gravity has been a fundamental force shaping the evolution of life on Earth, influencing various biological systems and functions. Organisms have adapted to Earth’s constant gravitational pull, and any deviation from this force can lead to significant physiological changes. In the context of space exploration, astronauts are subjected to varying gravitational forces, including microgravity and HyperG. Understanding how these altered gravity conditions affect human physiology is crucial for ensuring the health and performance of astronauts on current and future missions. In comparison to microgravity studies, there is limited insight into the cardiac effects of HyperG exposure. HyperG, defined as gravitational forces greater than Earth’s gravity (1G), is experienced by astronauts primarily during launch and re-entry, where forces can reach up to 8–9G [10,11]. Recent studies have shown that 4G exposure for 48 h disrupts calcium signaling in cardiomyocytes, activates CaMKII and HDAC4 pathways, and upregulates fetal genes such as ANP and BNP, all of which are markers of pathological cardiac remodeling [14]. Based on these findings, we developed a rat model exposed to 4G for 40 h to induce sustained, non-lethal cardiac stress. This protocol enables investigation of the initiation and progression of heart failure phenotypes under physiologically relevant conditions that impose substantial stress on the cardiovascular system.

However, due to the physical constraints of Earth’s gravity and the limited opportunities for space flight, researchers have turned to HyperG models to replicate these conditions in terrestrial settings. Given the physical impossibility to subtract from gravity on Earth, and the limited opportunities for space flight, many experimenters used the paradigm of HyperG to analyze the adaptation to altered gravity. The paradigm consists of adding a centrifuge force to the gravity, which is achieved by housing animals in a carousel whose gondolas are gimballed in such a way that the resultant force is perpendicular to the cage floor. The use of HyperG environments to study the alteration of gravity rests on the principle that the alteration of gravity acts as a continuum, based on the hypothesis formulated early in gravitational biology [15]. For example, previous research demonstrated that sustained HyperG exposure can impact muscle strength, bone density, and overall musculoskeletal health [16]. With reference to prior studies, the timeline of this study was adopted to observe delayed responses of the cardiovascular system after relatively short-term HyperG exposure.

There is limited direct evidence on the effects of HyperG exposure on cardiac fibrosis [17,18]. However, some studies have shown that cardiac fibrosis results from an imbalance between the production and degradation of the heart’s extracellular matrix (ECM), leading to cardiac dysfunction under pathophysiological conditions. In the process, TGF-β1 and MMP signaling pathways appear to play crucial roles in the development of cardiac fibrosis [19,20]. Excessive activation of TGF-β1 can promote the conversion of cardiac fibroblasts to myofibroblasts, potentially leading to cardiomyocyte death and increased cardiac stiffness [21,22]. In our study, we quantified protein levels of TGF-β1, MMP2, and MMP9 in rat hearts using Western blotting. These proteins were upregulated in HyperG-exposed rats and downregulated in rats receiving proton therapy. Contrary to this tendency, Connexin43 decreased in the HyperG group but was restored in the Combi group. These results demonstrate that proton therapy effectively inhibits cardiac fibrosis—likely through modulation of the TGF-β1 and MMP signaling pathways—while also preserving Connexin43 expression to maintain intercellular communication and cardiac conduction.

Calcium acts as a central intracellular messenger in the heart. CaMKII is activated through its binding protein calmodulin and regulates heart contraction, gene regulation, cell growth, and various cellular processes [23,24,25,26]. In our study, we observed activation of CaMKII in heart tissue exposed to HyperG, which decreased in rat heart tissue receiving proton therapy. In the context of heart disease, p-CaMKII plays a critical regulatory role in calcium handling by modulating key proteins such as RyR2 and PLB. p-CaMKII enhances the activity of RyR2, leading to increased sarcoplasmic reticulum (SR) calcium leakage, which can contribute to arrhythmias and cardiac dysfunction. Simultaneously, it phosphorylates PLB, altering calcium reuptake into the SR. Dysregulation of this signaling axis is implicated in pathological cardiac remodeling and the progression of heart failure. This suggests that proton therapy can regulate calcium homeostasis in the tissue. Additionally, heart gross images showed increased heart sizes in HyperG-exposed rats, which decreased in rats receiving proton therapy. These findings contribute to a better understanding of the effects of space flight and hypergravity exposure on heart function.

While direct research on AQP4 in astronauts’ cardiac function is limited, prolonged space flight can lead to decreased heart tissue contractility, increased arrhythmias, and higher mortality rates from cardiovascular diseases [27]. Additionally, in our previous study, we confirmed that increased AQP4 is associated with fibrosis and calcium-handling proteins in an atrial fibrillation mouse model [28]. AQP transcripts AQP1, AQP4, and AQP7 were identified in human and mouse hearts through real-time PCR and Northern blotting [29,30,31], and our study investigated the role of AQP4 in the negative impact of the space environment on the heart. Real-time PCR analysis showed significantly increased AQP4 mRNA levels in HyperG-exposed rats, with a tendency to recover in rats receiving proton therapy. Similar results were obtained at the protein level. Additionally, immunofluorescence detection of AQP4 confirmed higher expression in HyperG rat heart tissue compared to tissue receiving proton therapy. These findings may contribute to the development of new therapies related to maintaining heart health during space flight.

We report for the first time that proton therapy affects fibrosis, changes in calcium signaling, and water channels in the heart. Moreover, these changes may provide a basis for improving heart health in astronauts during long-duration space flights. Although our research is the first investigation to elucidate the effects of proton exposure on cardiac fibrosis, calcium homeostasis, and water transport channel AQP4, there are several limitations that need to be addressed for further study. First of all, only male rats were used since they tend to have more consistent physiological baselines compared to females, leading to more uniform data in controlled studies. Also, historical studies in space flight and HyperG research have used male rodents, making it suitable to compare results across other similar studies. Despite such reasons, conducting further research on female rats would allow for more generalization and identification of potential gender-specific effects of proton therapy. Secondly, we utilized the HyperG (4G)-exposed rat model to emulate the space environment. However, it has limitations in clinical application because it cannot fully reproduce the complexity of the actual space condition. Finally, in this study, experiments in terms of the specific part or proportion of the heart being irradiated were not performed. Also, this study focuses on fibrosis and calcium-handling proteins in heart tissue, but it is necessary to study the other organs exposed to proton therapy, as well as the relationship between HyperG and inflammation, in future studies. Furthermore, in this study, MMP2 and MMP9 were assessed exclusively by Western blot analysis, which precluded the precise quantification of their respective active forms. Therefore, to overcome such limitations, further research on cardiac function under optimized conditions and in-depth analysis at a cellular level will be necessary for practical application.

## 4. Materials and Methods

This study’s protocol was supported by the Institutional Animal Care and Use Committee of Inha University (IACUC-INHA231026-898) and conformed to the guidelines for the care and use of laboratory animals published by the United States National Institutes of Health.

### 4.1. Exposure of Hypergravity and Radiation on Animal Models

Male Sprague Dawley rats (12 weeks old) were acquired from Orient Bio Inc. (Seoungnam, Republic of Korea). A total of 20 rats were used with 5 rats per group. A subset of rats was exposed to hypergravity (HyperG) to induce cardiac hypertrophy. The rats in this study were subjected to HyperG (4G) over a 72 h period and then shipped to the Korea Multi-Purpose Accelerator Complex (KOMAC) to be irradiated. Rats were subjected to HyperG for 24 h a day (with a 1 h break for husbandry procedures) using our previously described custom-designed gravitational force (G-force) simulator [32]. In detail, the simulator consists of animal housing cages at the end of each of the two horizontal rotatory arms (50 cm long each). When the arms rotate at a speed of 57 rpm, the rats in the cage are exposed to 4G HyperG. A high-resolution video camera, which was placed inside the cage, was used to evaluate whether the rats could move freely and access food and water.

To ensure dose uniformity and to avoid attenuation of the 100 MeV proton beam within the body of the rats, protons were exposed only to the body of the rats. The rats were exposed to 10 cGy of 100 MeV proton linac generated at KOMAC at a dose rate of 9 cGy/min. The animal was positioned 1700 mm from the beam window. For the 100 MeV energy, measurements at the sample location showed an energy of 95.16 MeV. After recovery from the ketamine/xylazine, the rats were transported back to Inha University and, where appropriate, subjected to HyperG for an additional 72 h. All groups were sacrificed on the following day after the final exposure.

### 4.2. Histology Analysis and Immunofluorescence

In randomly selected rats, hearts were fixed in 4% paraformaldehyde (PFA) to preserve tissue morphology and prevent degradation. Following fixation, samples were processed through a graded series of ethanol, cleared in xylene, and embedded in paraffin using an automated tissue processor (TP1020, Leica Biosystems, Wetzlar, Germany). The resulting paraffin blocks were then sectioned in the sagittal plane at a thickness of 5 μm using a rotary microtome (Histocore Multicut R, Leica Biosystems). Sections were collected onto glass slides for subsequent analysis. For the assessment of myocardial fibrosis, Masson’s trichrome staining was performed using a commercial kit (Bioquochem, KH07007, Asturias, Spain) following the manufacturer’s instructions. Histological images were obtained on a slide scanner at 80× magnification (Olympus VS200, Tokyo, Japan). Quantification of the fibrotic area of the left ventricle was expressed as the percentage of stained areas in comparison with the total area of fields examined and was performed using ImageJ 1.52a image analysis software (National Institutes of Health, Bethesda, MD, USA).

For dual staining, sections were washed three times with PBS followed by antigen retrieval in 0.25% Triton for 10 min. Samples were blocked for 1 h in 10% goat serum followed by incubation with primary antibodies for 2 h at room temperature (RT). Antibodies against Troponin I (1/300, Abcam, Cambridge, UK, ab209809), AQP4 (1/100, Santa Cruz Biotechnology, Dallas, TX, USA, sc-32739), and Connexin 43 (1/100, Santa Cruz Biotechnology, sc-13558) were used. Alexa fluor 488 goat anti-mouse IgG (1:100, Abcam, ab150113) and Alexa fluor 594 goat anti-rabbit IgG secondary antibodies were used (1:100, Abcam, ab150080) for 1 h at RT. The slides were washed three times in PBS for 5 min each. Finally, the slides were mounted with mounting medium with DAPI (Aqueous, Fluoroshield, Abcam, ab104139). Slides were observed at 400× magnification using an inverted fluorescence microscope (Nikon, Ti2-U, Tokyo, Japan).

### 4.3. Western Blotting

An amount of 50 μg of total protein for each lane was separated using a 5% stacking gel and 10% separation SDS-PAGE gel and transferred to a 0.45 μm PVDF membrane (Millipore, Burlington, MA, USA). The membrane was incubated in TBST containing 5% nonfat milk for 2 h at room temperature to block nonspecific binding. The membrane was then incubated overnight with the primary antibody at 4 °C.

The primary antibodies against CaMKII (1:1000; Santa Cruz Biotechnology, sc-13082), p-CaMKII (1:1000; PA5-37833, Invitrogen, Carlsbad, CA, USA), RyR2 (1:1000; ab302716, Abcam, Cambridge, MA, USA), RyR2 p-Ser2814 (1:1000; A010-31-AP, Badrilla, Portland, UK), RyR2 p-Ser2808 (1:1000; Badrilla, A010-30-AP), PLB (1:1000; ABclonal, A23750), p-PLB (1:1000; EMD Millipore, 07-052), MMP9 (1:1000; Santa Cruz Biotechnology, sc-13520), TGF-β1 (1:1000; #3709, Cell signaling technology, Danvers, MA, USA), MMP2 (1:1000; Santa Cruz Biotechnology, sc-13595), Connexin43 (1:1000; Santa Cruz Biotechnology, sc-13558), AQP4 (1:1000; Santa Cruz Biotechnology, sc-32739), and the internal control antibody GAPDH (1:3000; Abcam, ab181602) were used. The membrane was incubated with the goat anti-mouse IgG HRP (1:5000, GenDEPOT, SA001-500) and goat anti-rabbit IgG HRP (1:5000, SA002-500, GenDEPOT, Katy, TX, USA) secondary antibodies (1:1000) for 1 h at room temperature. The membrane was incubated in chemiluminescent HRP substrate (Thermo Scientific, 34577, Waltham, MA, USA) at room temperature and then imaged with the ChemiDoc Imaging System (Bio-Rad, Hercules, CA, USA). Blots were scanned, and the band ratio and intensity were quantified using Image J 1.52a software.

### 4.4. Statistical Analysis

Data were expressed as the means ± SEMs. ANOVA tests with post hoc and Bonferroni’s corrections were used to compare the means among the four groups. Pearson’s chi-squared tests were used to compare two categorical variables. Analysis was performed using the statistical software package SPSS version 23.0 (IBM^®®^, Armonk, NY, USA). A *p*-value < 0.05 was considered statistically significant.

## 5. Conclusions

Our study investigates the effects of HyperG exposure and proton therapy on cardiac fibrosis, calcium handling, and AQP4 expression in rats. HyperG exposure led to increased heart weight, fibrosis, disrupted calcium signaling, and elevated AQP4 expression. On the other hand, proton therapy reduced these effects by downregulating fibrosis-related proteins (TGF-β1, MMP2, MMP9), restoring calcium homeostasis, and decreasing AQP4 expression. These findings highlight the potential of proton therapy in mitigating heart dysfunction caused by extreme environments, offering insights into astronaut health during space travel.

## Data Availability

The original contributions presented in this study are included in the article. Further inquiries can be directed to the corresponding author.

## Abbreviations

Hypergravity—HyperG, Combination—Combi, Ca^2+^/calmodulin-dependent protein kinase II—CaMKII, Phospholamban—PLB, Ryanodine 2 receptor—RyR2.
